# Field and experimental symptomless infections support wandering donkeys as healthy carriers of *Trypanosoma vivax* in the Brazilian Semiarid, a region of outbreaks of high mortality in cattle and sheep

**DOI:** 10.1186/s13071-015-1169-7

**Published:** 2015-10-28

**Authors:** Carla MF Rodrigues, Jael S. Batista, Joseney M. Lima, Francisco JC Freitas, Isabella O. Barros, Herakles A. Garcia, Adriana C. Rodrigues, Erney P. Camargo, Marta MG Teixeira

**Affiliations:** Departamento de Parasitologia, Instituto de Ciências Biomédicas, Universidade de São Paulo, São Paulo, SP Brazil; Departamento de Ciências Animais, Universidade Federal Rural do Semi-Árido, Mossoró, Rio Grande do Norte Brazil; Colegiado de Medicina Veterinária, Universidade Federal do Piauí, Bom Jesus, Piauí Brazil; Departamento de Patología Veterinaria, Facultad de Ciencias Veterinarias, Universidad Central de Venezuela, Maracay, Aragua Venezuela

**Keywords:** *Trypanosoma vivax*, Donkey, Reservoir, PCR-diagnosis, Trypanotolerance, Molecular epidemiology, Genotyping, South America

## Abstract

**Background:**

The Brazilian Semiarid is the home of the largest herd of donkeys in South America and of outbreaks of *Trypanosoma vivax* infection of high mortality in dairy cattle and sheep. For a comprehensive understanding of the underlying mechanisms of these outbreaks and epidemiological role of donkeys, we surveyed for *T. vivax* in wandering donkeys and follow the experimental infection of donkeys and sheep with a highly virulent isolate from the Semiarid.

**Methods:**

Blood samples from 180 randomly selected wandering donkeys from the Brazilian Semiarid region were employed for PCV and parasitemia assessments and tested using the *T. vivax*-specific TviCATL-PCR assay. PCR-amplifed Cathepsin L (CATL) sequences were employed for genotyping and phylogenetic analysis. Four wandering donkeys were experimentally infected with a *T. vivax* isolate obtained during an outbreak of high mortality in the Semiarid; the control group consisted of two non-inoculated donkeys.

**Results:**

We detected *T. vivax* in 30 of 180 wandering donkeys (16.6 %) using TviCATL*-*PCR. The prevalence was higher during the dry (15.5 %) than the wet season (1.1 %) and more females (23.1 %) than males (8.9 %) were infected*.* All the PCR-positive donkeys lacked patent parasitemia and showed normal values of body condition score (BCS) and packed cell volume (PCV). To evaluate the probable tolerance of donkeys to *T. vivax*, we inoculated five donkeys with a highly virulent isolate (TviBrRp) from the Semiarid. All inoculated donkeys became PCR-positive, but their parasitemia was always subpatent. A control goat inoculated with TviBrRp showed increasing parasitemia concurrently with fever, declining PCV, tachycardia, mucous membrane pallor, enlarged lymph nodes and anorexia. None of these signs were observed in donkeys. However, *T. vivax* from wandering donkeys shared identical or highly similar genotypes (identified by Cathepsin L sequences) with isolates from cattle and sheep outbreaks of acute disease in the Semiarid.

**Conclusions:**

This is the first report of *T. vivax* in donkeys in Brazil and, to our knowledge, the first experimental infection of donkeys with *T. vivax*. The symptomless field and experimental infections corroborated that donkeys are more tolerant to *T. vivax* than other livestock species as shown in African countries*.* Therefore, farmers, veterinaries and control programmes should be aware of healthy carrier donkeys as a possible source of *T. vivax* for susceptible livestock species in the Brazilian Semiarid.

**Electronic supplementary material:**

The online version of this article (doi:10.1186/s13071-015-1169-7) contains supplementary material, which is available to authorized users.

## Background

In endemic sites of South America, beef cattle and water buffaloes infected with *T. vivax* are mostly symptomless and lack patent parasitemias. In Brazil, asymptomatic infection of beef cattle and water buffaloes, mostly detectable exclusively by PCR, has been reported in Amazonia and the Pantanal [1–3], despite a few outbreaks of disease in cattle that occurred approximately 20 years ago in the wetlands of Brazil and Bolivia [4, 5]. Because livestock in these regions are co-infected with many other parasites, the role of *T. vivax* in clinical and pathological manifestations is questionable. The maintenance of *T. vivax* in the enzootic areas depends on the abundance of both biting flies and prevalent infected animals warranting the mechanical transmission of the parasite. Although tabanids are implicated as the main mechanical vectors of *T. vivax*, *Haematobia irritans* and *Stomoxys calcitrans* have also been considered possible vectors. In addition, contaminated needles have largely contributed to *T. vivax* transmission in Brazilian outbreaks [6, 7].

Outbreaks of severe acute infections by *T. vivax* were reported in dry cattle throughout the Brazilian territory in recent years. The successive outbreak reports from distantly separated regions suggested that *T. vivax* is presently widespread in former non-endemic regions of Brazilian Semiarid where outbreaks have been reported in dairy cattle and sheep. Although serological surveys suggested that *T. vivax* was not disseminated in cattle herds in areas where previous outbreaks were controlled by the treatment of all symptomatic animals [8, 9], the answer to this question requires extensive surveys using sensitive diagnostic methods in healthy goats, horses and donkeys, which are carriers of *T. vivax* in Africa [10–12].

In the Semiarid, the infection of livestock with *T. vivax* has resulted in marked failures in productivity due to the associated progressive anaemia, weight loss, death, abortion, perinatal mortality, pregnancy decline and drop in milk production, significantly reducing the livestock productivity over short and long time scales [8, 13–15]. We previously demonstrated that in the Brazilian Semiarid, goats that spontaneously recovered from acute infection can develop chronic disease that can be reactivated by stressing conditions during long and very hot and dry seasons [13]. The possibility that goats, which are highly abundant in the Semiarid, can be healthy carriers of *T. vivax* deserves a broad investigation. In Africa, *T. vivax* can be pathogenic in bovines and small ruminants, causing chronic and progressive anaemia and, rarely, disseminated haemorrhagic syndrome [16, 17]. Studies of *T. vivax* in African donkeys are mostly from Ethiopia, which is the home of ~6 million donkeys, with few reports from the Gambia, Kenya, Sudan and Burkina Faso, where trypanosomosis is a major constraint for livestock production in both tsetse-infested and tsetse-free areas. In these countries, *T. vivax* causes symptomless or mild infections in donkeys, whereas cattle can develop debilitating disease [10, 11, 17–20].

African wild ruminants, such as buffaloes and antelopes, are reservoirs of *T. vivax* in Sub-Saharan Africa [21, 22], a role that can be played by healthy donkeys and some breeds of goats in the Sahelian region [10–12]. The existence of sylvatic reservoirs of *T. vivax* is an open question in South America, but beef cattle and water buffaloes are common healthy carriers. In the endemic regions, these animals can exhibit low PCV when co-infected with *T. vivax, Babesia* spp and *Anaplasma marginale,* which are hemoparasites known to induce severe anaemias and to be predisposing factors for the infection of bovines with *T. vivax* in South America [3, 23–27]*.*

Despite very low parasitemias, when livestock from areas endemic for *T. vivax* are introduced into naïve herds and biting flies are abundant, these animals serve as effective sources of parasites, triggering a slow initial infection of a few animals that serve as sources of parasites for outbreaks of rapid propagation. Cattle and buffaloes imported from endemic areas have been tracked as the sources of *T. vivax* for some outbreaks in livestock production areas in Brazil. However, it was impossible to track animals from endemic areas imported into many outbreak sites in the Semiarid, suggesting that local animals might have assumed the role of reservoirs, and donkeys are good candidates for healthy carriers of *T. vivax* in the Brazilian Semiarid.

The goals of this paper were to verify whether, similar to Africa, donkeys in the Brazilian Semiarid are infected with *T. vivax* and can act as asymptomatic carriers of the parasite. This is a highly relevant question to the epidemiology of disease outbreaks in this region. For a comprehensive understanding of the underlying mechanisms of these outbreaks and epidemiological role of donkeys, we employed parasitological, molecular and clinical analyses to survey *T. vivax* in wandering donkeys and to follow the course of the experimental infection of donkeys with a highly virulent *T. vivax* isolate from the Semiarid.

## Methods

### Study area, donkeys and sampling strategy

**Fig. 1:**
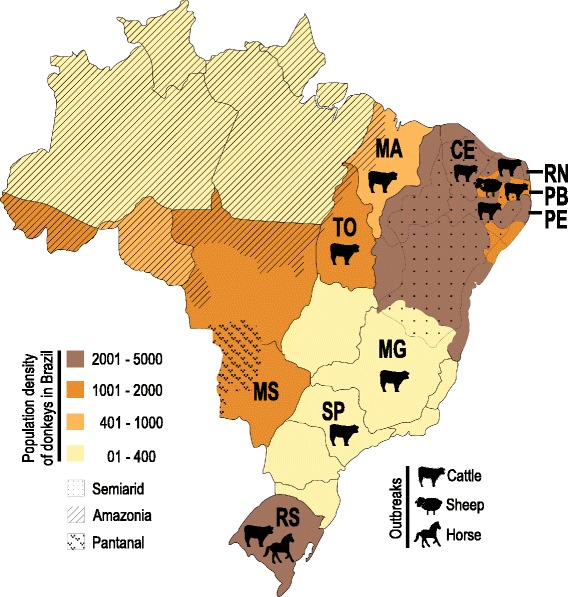
Map of Brazil showing donkey populations and the sites of outbreaks of *T. vivax* infection. Outbreaks in dairy cattle, sheep and horses occurred in the States of Rio Grande do Norte (RN), Paraiba (PB), Pernambuco (PE), Ceará (CE), Maranhão (MA), Tocantins (TO), Minas Gerais (MG), São Paulo (SP) and Rio Grande do Sul (RS). The Brazilian *T. vivax* endemic regions are in The Pantanal and Amazonian wetlands

This study was conducted in the city of Mossoró, State of Rio Grande do Norte (RN) (S 5–6^0^, W 36–38^0^) in the Brazilian Semiarid region (Fig. 1). This tropical region is predominated by the unique Caatinga Biome, which has a hot and dry climate with recurrent droughts and is characterized by xerophytic shrubs and small trees, often showing thorns and deciduous leaves. The vegetation of this biome includes many cacti, bromeliads, grasses, legumes and forbs. Little and irregular rainfall hamper the production of broad crops, and small or subsistence crops characterize this region. Livestock production, mainly goats and sheep, is the most important economic activity in the Brazilian Semiarid. During the rainy season, most forage resources are from the herbaceous stratum, whereas the leaves from woody species become the main food for ruminants in the dry season [28]. The average temperature in the Semiarid is 27.5 °C (22 °C - 34 °C). The short rainy season begins in January and ends in May, with an average rainfall of ~150 mm (~100 to 180 mm) and a mean relative humidity of 75 %. Some very dry years have only a few days of rainfall, which mostly occur in April. The long dry season extends from June to the end of December with low (22 °C) and high (35 °C) temperature mean values. Rainfalls drastically decreased during the dry season; in the driest period between August and November, the rainfall ranges from ~11 to < 3.0 mm with a mean relative humidity of ~60 % (http://www.inmet.gov.br/portal/). Haematophagous biting flies are scarce during the dry season but can be abundant during wet periods [6, 8, 13].

Donkeys (*Equus asinus*) in the Semiarid (“Nordestino’ breed) usually share pastures with other livestock in small farms. Wandering donkeys, found everywhere in the area studied herein, have been captured and held in custody by the Highway Federal Police, who provided the animals for this study. From August 2010 to April 2011, 180 male and female donkeys of various ages and BCSs and including male and females were randomly selected among the donkeys captured 5–7 days prior; 79 and 101 donkeys, respectively, were examined during the dry (June-December ) and wet (January-May) seasons (Additional file 1). Approximately 2.0 ml of blood from each animal was collected by puncture of the jugular vein into tubes containing 1.0 mg/ml EDTA, submitted to microscopy and microhaematocrit (MH) technique [29] and used for DNA preparations.

### Experimental infection of donkeys and a goat with *T. vivax*

For experimental infection, in January 2013 we randomly selected seven donkeys (~18 months of age) among the donkeys kept in the custody centre in Mossoró (RN). The animals were housed in a screened picket at the Rural University of the Semi-Arid (UFERSA, Mossoró, RN) for clinical evaluation for 7 days before inoculation. Through the experimental period, the animals were fed daily with tifton hay (*Cynodon*) supplemented with 1.5 % of their body weight of commercial food and water ad libitum. Blood samples were collected from the donkeys immediately upon arrival and 7 days afterward. All animals were clinically healthy, with normal PCV and rectal temperature (RT), absence of *T. vivax* parasitemia by microscopy and MH and negative results for TviCATL-PCR, excluding one donkey that was weakly positive by PCR.

The donkeys were distributed into two experimental groups. One group of inoculated animals consisted of four donkeys that were PCR-negative for *T. vivax* (#1 and #2 males, #3 and #4 females) plus the donkey that was PCR-positive (#5 female). The control group was represented by two non-inoculated donkeys (#6 male and #7 female). In addition, one goat from the same area of RN state was also inoculated to serve as a positive control. The TviBrRp isolate of *T. vivax* used for the inoculation was obtained from a sheep infected in an outbreak in São João do Rio do Peixe, in the neighbouring State of Paraiba (PB), which died with severe haematological and nervous perturbations [6]. Blood samples from an experimentally infected goat cryopreserved in liquid nitrogen were thawed and microscopically examined for parasite viability before inoculating the animals. Each animal received an intravenous inoculum of 1.25 × 10^5^ trypomastigotes.

### Ethical approval

The design and methodology of all the experiments involving donkeys and goats were conducted in accordance with the guidelines of the Brazilian College of Animal Experimentation, following the Brazilian law for “Procedures for the Scientific Use of Animals” (11.794/2008). The Animal Care Ethics Committee of the UFERSA (RN) (23091.003209/2011-10) and ICB-USP (Protocol n° 009, page 3 of book 3) approved the study.

### Anemia, parasitemia and clinical assessments

For 30 days post-infection (dpi), animals from the infected and control groups were clinically examined to assess their RT, heart and respiratory rates, skin and eye abnormalities, mucous membrane coloration and external lymph node volume. The BCS was determined weekly on a scale from zero to five for skinny to fat animals, and parasitemia was determined daily using blood collected from the ear (100 ul) by microscopic analyses in MH capillary and fresh blood examination [8, 13]. Blood samples were collected by puncture of the jugular vein for PCV analysis, as an indicator of anaemia, and for DNA preparations using blood samples collected at 1, 5, 10, 15, 20 and 30 dpi.

### Statistical analyses

The prevalence of *T. vivax* was examined to assess if the presence of parasite in wandering donkeys was associated with age, PCV and BCS using the Chi-square (χ2) test of independence, whereas associations of prevalence with sex and season (dry and wet) were assessed by the Fisher’s exact test. Statistical analyses were done using the BioEstat v5.0 software and significance was accepted at the 95 % confidence level. Tukey’s test was used to compare the means of parasitemia, PCV, RT and BCS indexes between *T. vivax* experimentally infected and control non-infected donkeys using the SAS statistical software package. The results were considered significant at *p* < 0.05.

### Molecular diagnosis, genotyping and phylogenetic analysis

Blood samples from donkeys preserved in ethanol (200 μl) were processed for the preparation of DNA samples [21] used as templates for the sensitive PCR (TviCATL-PCR) specific to *T. vivax* and amplifying a ~177 bp fragment of the multicopy Cathepsin L-like gene [30]. The DNA of the isolate TviBrRp served as positive control. The PCR products were electrophoresed in 2.0 % agarose gels and stained with ethidium bromide. PCR-amplified bands were excised from the gel and sequenced. CATL-amplified DNA were cloned, and 5–7 clones from selected samples were sequenced and aligned with sequences of *T. vivax* isolates from the nearby RN and PB states and other South American isolates [30, 31]. The alignment including sequences of *T. vivax* isolates from Brazil, Venezuela, The Gambia, Burkina Faso, Nigeria, Mozambique, Kenya and Zambia [30, 31] was employed for neighbour joining phylogenetic analysis as described previously [32].

DNA from the donkey blood samples was also tested by PCR aiming to investigate *T. evansi* infection (the method is able to detect all *Trypanozoon* species) as previously reported [3].

## Results and discussion

### *T. vivax* in donkeys from the Brazilian Semiarid

**Fig. 2:**
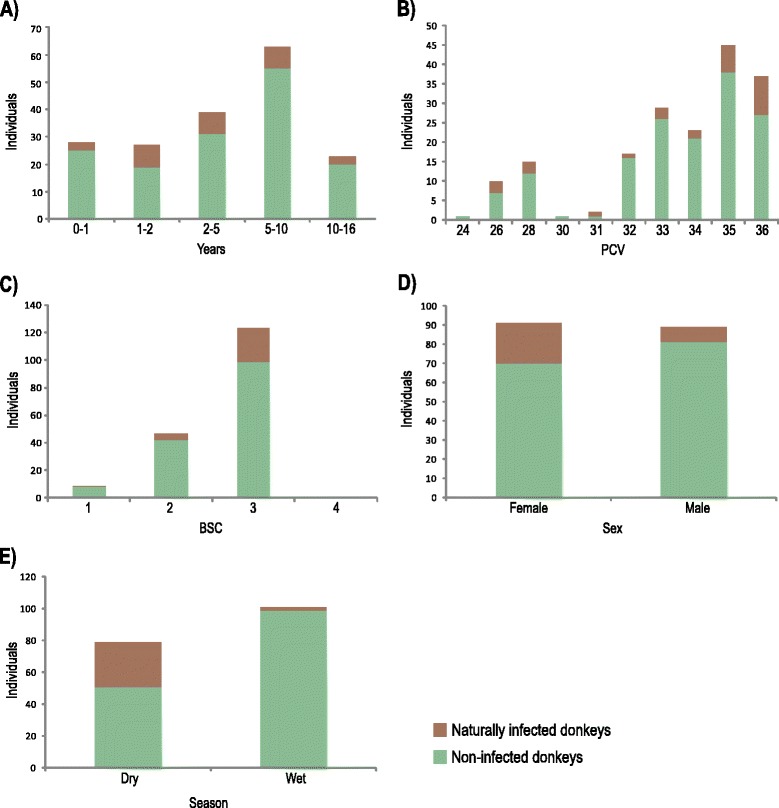
Epidemiological survey of *T. vivax* in wandering donkeys from the Brazilian Semiarid using TviCATL-PCR. Association analyses of *T.vivax* prevalence in wandering donkeys from the state of Rio Grande do Norte (RN) was not statically significant with **a** age (years), **b** packed cell volume (PCV) and **c** body condition score (BCS) but was with **d** sex (p = 0.014) and **e** season (dry and wet months) when donkey blood samples were collected (p = 0.000)

We examined blood samples from 180 wandering donkeys captured on the roadways of the RN State in the Brazilian Semiarid. All samples were negative by MH and microscopic analyses. In contrast, species-specific TviCATL-PCR [30] revealed 30 donkeys (16.6 %) that were positive for *T. vivax* (Fig. 2; Additional file 1)*.* Although the blood samples from several animals generated weakly amplified DNA bands, positive results were corroborated by additional PCRs using DNA preparations from other aliquots of donkeys blood samples. Sequencing of amplified DNA from selected samples (~25 %) confirmed the diagnosis*.* In previous studies, the most sensitive method used to detect *T. vivax* in donkeys consisted of initial PCR for whole genome amplification (WGA) followed by species-specific PCR, thus increasing the detection level of *T. vivax* above the threshold of any conventional PCR [11].

No association was detected between positivity for *T. vivax* and donkey PCV, BCS or age (Fig. 2). *T. vivax* was detected by PCR in the youngest (3–6 months) and oldest (7–16 years) donkeys examined. In addition, most infected animals showed normal BCS of 3 (maximum BCS was 4 for donkeys in the studied area). The low BCS (<3) exhibited by few animals could not be related to *T. vivax* infection or enhanced parasitemia. More females (23.1 %, 21 of 91) than males (8.9 %, 8 of 89) were infected (*p* = 0.014) (Fig. 2). Pregnant and lactating animals could not be linked to the large number of female infected with *T. vivax*. There was a marked significant difference (*p* = 0.000) in the prevalence of *T. vivax* in donkeys between the dry (15.5 %, 28 of 79 donkeys examined) and the wet (1.1 %, 2 of 101 donkeys examined) seasons (Fig. 2).

We did not detect donkeys that were PCR-positive for *T. evansi* in The Brazilian Semiarid*,* the commonest and highly pathogenic species for equines also exclusively mechanically transmitted and common in areas endemic for *T. vivax* in Brazil [3, 33]. In Venezuela, donkeys of endemic settings are known to be healthy carriers of *T. evansi* [34].

### Symptomless donkeys infected with virulent *T. vivax* from the Brazilian Semiarid

**Fig. 3:**
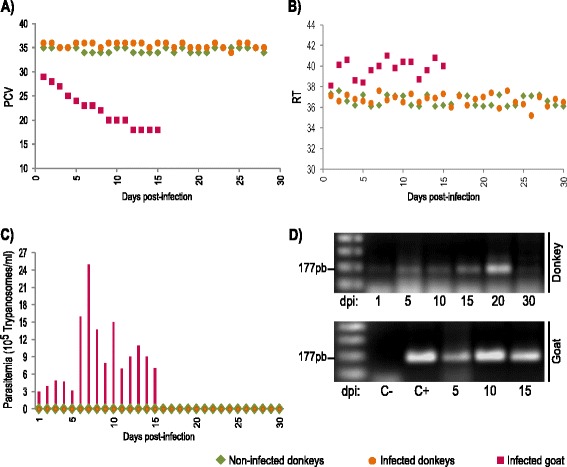
Experimental infection of five donkeys and one goat with the *T. vivax* isolate TviBrRp. The parameters evaluated daily in the infected and non-infected donkeys over 30 experimental days were: **a** packed cell volume (PCV); **b** rectal temperatures (RT) in °C; **c** parasitemia (×10^5^ trypanosomes/ml). **d** Illustrative figure shown to illustrate the general results of detection of *T. vivax* using the method of TviCATL-PCR (amplified DNA bands of ~177 bp resolved in agarose gels and stained with ethidium bromide) in blood samples of donkeys and a control goat (susceptible animal) following the course of infection from the 1st to the 30th day post-infection (dpi)

To complement the field information regarding the apparent donkey resistance to *T. vivax*, we studied the clinical course of infection in donkeys that were experimentally infected with the highly virulent *T. vivax* isolate TviBrRp. MH and blood smears did not reveal trypanosomes in any of the experimentally infected donkeys during the 30 days of observation. However, the TviCATL-PCR results were positive for all of the inoculated animals. The intensity of PCR bands at the 20th dpi was stronger than the weaker bands detected before, indicating parasite multiplication, then DNA bands became very faint until the end of experiment (Fig. 3).

The *T. vivax*-inoculated donkeys were examined daily with respect to PCV values, RT, breathing and heart rates, mucosa pallor, external lymph nodes and BCS. These parameters remained normal for both inoculated (#1–5) and control donkeys (#6–7) until the end of experimental study. The donkey (#5) that was positive by PCR before the experimental infection was also inoculated with *T. vivax* and did not display significant differences in the course of infection compared to those of other donkeys. We could not rule out the possibility that the PCR-negative donkeys had also been previously infected with *T. vivax* and thus developed a protective immune response against acute infection. To clarify this issue, it will be necessary to verify whether donkeys from areas that are free of *T. vivax* develop parasitemic and symptomatic infections when experimentally infected.

In contrast to all of the inoculated donkeys, the control goat showed high parasitemia and strong PCR bands; the animal became very sick and was euthanized at the 15th dpi. The infected goat presented all of the signs of a severe infection: high RT, rapidly decreasing PCV, marked weakness, accelerated heart rate, abnormal respiratory rate, enlarged lymph nodes and mucous membrane pallor (Fig. 3). All these findings are consistent with previous reports of acute infection with TviBrRp in goats and sheep, revealing typical waves of parasitemia and evolving to severe anaemia and nervous and reproductive disorders after 30 days of infection [6, 14, 15, 35].

To our knowledge, this is the first experimental infection of donkeys with *T. vivax*. The symptomless field and experimental infections corroborated that donkeys are more tolerant to *T. vivax* than other livestock species as shown in African countries [10, 11, 17–20]*.*

### Genotyping of *T. vivax* from donkeys, cattle and sheep from the Brazilian Semiarid

Increasing repertoires of *T. vivax* genotypes have been disclosed by comparing CATL sequences. Phylogenetic relationships inferred with CATL sequences of *T. vivax* were highly congruent with those generated by SSU rRNA, gGAPDH and ITS rDNA sequences [1, 21, 30, 31], *T. congolense* [32] and *T. theileri* [36, 37]. Here, the CATL DNA fragments amplified by TviCATL-PCR [30] from *T. vivax* field-infected donkeys were cloned, sequenced, and sequences determined for 5–7 clones of each donkey. Sequences from blood of two donkeys infected with *T. vivax* were compared with those obtained from cattle and sheep outbreaks that had occurred in nearby farms [6, 8].

**Fig. 4:**
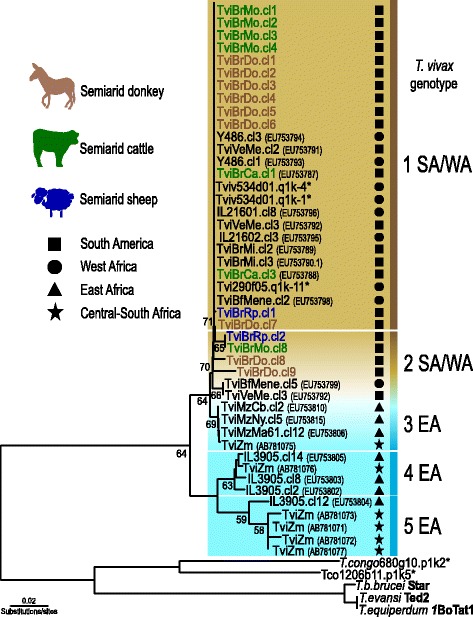
Phylogeny of *T. vivax* cathepsin L (CATL) gene sequences of wandering donkeys from the Brazilian Semiarid. CATL sequences amplified using TviCATL-PCR from blood samples of donkeys (TviBrDo) were aligned to sequences from isolates of sick dairy cattle (TviBrMo and TviBrCa) and sheep (TviBrRp) outbreaks of the Semiarid; sequences of *T. vivax* infecting livestock in endemic South American and African countries were included in the alignment. Numbers at the nodes are bootstrap support values from 500 replicates.*http://www.sanger.ac.uk/Projects/T_vivax/. Codes within parentheses are GenBank accession numbers

CATL sequences of *T. vivax* isolates from the wandering donkeys (TviBrDo) were identical or highly similar to those from isolates of outbreaks of acute disease reported in the last 10 years in cattle (TviBrCa and TviBrMo) and sheep (TviBrRp) in the Semiarid, and highly similar to *T. vivax* of symptomless animals from endemic regions (Fig. 4). Therefore, symptomless donkeys and very sick sheep and dairy cattle living in the Semiarid can be infected by the same genotypes. In Brazil, as we previously suggested by comparing isolates from endemic (enzootic) and non-endemic (outbreaks) settings, clinical manifestations are dependent on prior contact with *T. vivax* and the degrees of tolerance of the species and breeds of livestock, and are not related to a particular genotype of *T. vivax* [1]*.*

Phylogenetic analysis were inferred including sequences representative of the small genetic diversity of CATL sequences obtained from donkeys, all corresponding to genotypes previously reported for South American and West African *T. vivax,* whereas East and Central-South African isolates exhibited distinct genotypes, with the exception of some sequences from Mozambique and Zambia that were closely related to South American genotypes (Fig. 4). As expected, isolates from Brazil were more closely related to those from West Africa. In addition, we provided new evidence that the East African population is composed of genotypes that can be either closely or distantly related to the West African and South American genotypes as suggested in previous studies [1, 21, 30, 31].

Unfortunately, attempts to amplify ITS rDNA sequences from *T. vivax* in donkey blood samples failed (data not shown), reinforcing previous studies demonstrating that PCR targeting ITS rDNA has a low sensitivity [22, 38]. The DNA sequences targeted by other PCR methods valuable for the diagnosis of *T. vivax* require evaluation for genotyping purposes [11, 39]. Microsatellite analysis revealed that very similar but different genotypes of *T. vivax* circulate in the Semiarid and other Brazilian regions [1]. Unfortunately, our attempts to include samples from donkeys in the microsatellite analysis were unsuccessful.

### Donkeys as healthy carriers in the epidemiological scenario of *T. vivax* infection in the Brazilian Semiarid

We demonstrated that *T. vivax*-infected wandering donkeys of the Semiarid lacked patent parasitemia and clinical signs, thus playing a role as healthy carriers. The prevalence detected by TviCATL PCR was largest in the dry season, when donkeys are subjected to stressful conditions due to prolonged periods of drought and scarcity of forage and water. TviCATL PCR is a sensitive PCR method that permitted the detection of sub-patent parasitemias [1, 30, 31, 40–43]. The donkeys positive for *T. vivax* by PCR were likely carrying a chronic infection acquired in the wet season when abundant flies favour the transmission of parasites. Outbreaks in the Semiarid have been associated with high dairy cattle and sheep herd densities, high temperatures and atypically higher rainfalls, altogether prompting greater reproduction of flies and faster dissemination of *T. vivax* [6, 8]. However, an outbreak occurred in southeast Brazil during a dry and hot period on a farm where biting flies proliferated intensively in the waste from sugar and ethanol production [7]. In Sahelian Africa, donkeys show a high prevalence of chronic infections that favour their role as carriers of *T. vivax*, which is endemic throughout Africa. Endemic regions are characterized by the presence of reservoirs with subclinical infections and very low parasitemia, in general, detectable exclusively by molecular diagnosis. Previous studies demonstrated that the prevalence of *T. vivax* in African donkeys varies according to the country/region, season and transmission (cyclical by tsetse or mechanical by other hematophagous flies), and especially to the sensitivity of the diagnostic method employed to detect *T. vivax* infection [10–12, 44].

In the Brazilian Semiarid, we previously reported a few *T. vivax*-infected goats, sheep and cattle lacking apparent symptoms (diagnosed by PCR) on farms on which outbreaks of high mortality had been controlled by treatment restricted to sick animals [1, 6, 13, 14]. The treatment of acutely infected cattle, sheep and horses with diminazene aceturate induces an effective clearance of parasites (and DNA from dead parasites) from the blood, as confirmed by negative CATL-PCR results. However, relapses, detectable by CATL-PCR before parasites are detectable by parasitological methods, are common in animals exhibiting neurological disorders before the treatment, which may be associated with the extravascular migration of the parasite, and these animals often did not respond to treatment and died [6, 8, 14, 41].

Symptomless and low parasitemic *T. vivax* infection in wandering donkeys were corroborated by the lack of clinical signs in donkeys experimentally infected with a highly virulent isolate of *T. vivax* from the Brazilian Semiarid. In contrast, we previously showed that dairy cattle and sheep are highly susceptible to *T. vivax* infection [6, 8, 13]. Trypanotolerance is an innate genetic mechanism that refers to the capacity to tolerate the effects of trypanosome infections by remaining healthy under natural and experimental infections without treatment. *T. vivax*-infected horses were reported in an outbreak of high mortality in Southern Brazil [41]. In accordance with the African studies, our findings suggested that Brazilian donkeys are more tolerant to *T. vivax* than other livestock species, even horses [10, 11, 18, 41, 44, 45].

## Conclusions

This is the first report of *T. vivax* infecting wandering donkeys in the Brazilian Semiarid, a region of outbreaks of high mortality in dairy cattle herds and sheep flocks. To our knowledge, it is the first time that donkeys were found infected in Brazil and the first report of donkeys experimentally infected with *T. vivax*. Field and experimental symptomless infections support wandering donkeys as healthy carriers of *T. vivax*. Epidemiological and experimental evidences suggested a high tolerance of donkeys to *T. vivax* consistent with data reported in Africa and with the marked contrasting clinical evolution of tolerant donkeys and susceptible goat that developed fatal cute infection when infected with the highly virulent isolate TviBrRp. In addition, in the Semiarid, donkeys sharing pastures with dairy cattle and sheep during outbreaks of high mortality in the Semiarid never developed symptomatic infection.

Due to symptomless infections and low parasitemia, donkeys can remain undiagnosed despite their potential role in the maintenance of *T. vivax* in the Brazilian Semiarid. Their parasitemia can vary according to the health conditions, levels of nutritional and work stress, pregnancy, concurrent infections and season. Although donkeys are highly adapted to adverse conditions, during the very hot and dry periods (droughts lasting for longer than 1 year are common in the Brazilian Semiarid), limitations of food and water intake can likely reduce the ability of chronically infected wandering donkeys to control the parasitemia. Seasonal immunosuppression promoting increased parasitemia in healthy carriers of *T. vivax* and vector proliferation triggering new outbreaks appears to be typical of mechanically transmitted *T. vivax* in South America [5, 13, 24]. In aparasitemic infections, trypanosomes can be found in extravascular foci, such as lymph nodes, the aqueous humour of the eyes and cerebrospinal fluid. Stress and concomitant infections can trigger parasite reactivation and relapses of parasitemia. Reactivation can be experimentally demonstrated by food restriction and immunosuppressive treatment of low parasitemic livestock [5, 24, 46].

Although *T. vivax* transmission from donkeys or other low parasitemic healthy carriers to livestock was not accomplished under field experimental conditions in Brazil or in African countries, donkeys may act as a possible source of *T. vivax* for susceptible livestock species. Based on our findings, farmers, veterinaries and authorities in charge of the management of wandering donkeys and control programmes of outbreaks should be aware of the role of donkeys as healthy carriers of *T. vivax* and, hence, as possible sources of parasite dispersion and infection of susceptible livestock in the Brazilian Semiarid.

## Additional file

Additional file 1:
**Table showing sex, age, season of blood collection, TviCatl PCR results, body score condition (BSC) and packed cell volume (PCV) of the 180 donkeys examined in this study.** (PDF 322 kb)
