# Supplementary material for: Field and experimental symptomless infections support wandering donkeys as healthy carriers of Trypanosoma vivax in the Brazilian Semiarid, a region of outbreaks of high mortality in cattle and sheep
Source: Parasit Vectors. 2015 Oct 28;8:564. doi: 10.1186/s13071-015-1169-7 (PMC4625931; doi:10.1186/s13071-015-1169-7)
Supplement: Additional file 1: — Table showing sex, age, season of blood collection, TviCatl PCR results, body score condition (BSC) and packed cell volume (PCV) of the 180 donkeys examined in this study. (PDF 322 kb) [file 13071_2015_1169_MOESM1_ESM.pdf]

Additional file 1

Sex, age, season of blood collection, TviCatl PCR result, body score condition (BSC) and packed cell volume (PCV) of donkeys examined in this study

Donkey identification	Sex	Age	Collection season	TviCatL PCR	BSC	PCV
1	Female	2-5	Dry	Positive	3	28
2	Female	2-5	Dry	Negative	3	33
3	Female	2-5	Dry	Positive	3	36
4	Female	1-2	Dry	Positive	3	35
5	Female	1-2	Dry	Positive	3	33
6	Female	5-10	Dry	Positive	3	28
7	Female	>10	Dry	Positive	3	35
8	Male	1-2	Dry	Positive	3	34
9	Female	2-5	Dry	Positive	3	32
10	Female	5-10	Dry	Positive	2	26
11	Female	5-10	Dry	Positive	2	36
12	Female	0-1	Dry	Negative	3	36
13	Female	2-5	Dry	Positive	3	35
14	Male	0-1	Dry	Negative	3	33
15	Female	5-10	Dry	Positive	3	35
16	Female	2-5	Dry	Positive	3	36
17	Female	>10	Dry	Negative	3	31
18	Female	1-2	Dry	Positive	3	36
19	Female	5-10	Dry	Negative	2	36
20	Male	>10	Dry	Positive	1	35
21	Male	1-2	Dry	Negative	3	33
22	Male	2-5	Dry	Negative	3	36
23	Female	1-2	Dry	Positive	3	36
24	Male	5-10	Dry	Positive	3	31
25	Male	5-10	Dry	Negative	3	36
26	Female	2-5	Dry	Negative	3	36
27	Female	2-5	Dry	Positive	2	34
28	Female	5-10	Dry	Negative	3	32
29	Female	5-10	Dry	Positive	3	26
30	Female	0-1	Dry	Positive	3	36
31	Male	2-5	Dry	Positive	3	36
32	Male	2-5	Dry	Negative	4	35
33	Female	>10	Dry	Positive	2	33
34	Male	0-1	Dry	Positive	2	36
35	Female	2-5	Dry	Negative	3	36
36	Female	5-10	Dry	Negative	3	35
37	Male	1-2	Dry	Positive	3	33
38	Male	2-5	Dry	Positive	3	36
39	Female	1-2	Dry	Positive	3	35
40	Male	0-1	Dry	Negative	3	33
41	Female	5-10	Dry	Negative	3	28
42	Female	5-10	Dry	Negative	3	35
43	Female	5-10	Dry	Negative	3	34
44	Male	1-2	Dry	Negative	3	32
45	Female	5-10	Dry	Positive	3	26
46	Male	2-5	Dry	Negative	3	35
47	Male	1-2	Dry	Negative	3	33
48	Male	>10	Dry	Negative	1	36
49	Male	5-10	Dry	Negative	3	35
50	Male	>10	Dry	Negative	3	33
51	Male	2-5	Dry	Negative	3	34
52	Female	2-5	Dry	Negative	3	32
53	Female	>10	Dry	Negative	3	26
54	Female	0-1	Dry	Negative	1	36
55	Male	>10	Dry	Negative	2	36
56	Male	5-10	Dry	Negative	2	35
57	Male	>10	Dry	Negative	3	33
58	Male	>10	Dry	Negative	3	28
59	Male	0-1	Wet	Negative	3	35
60	Male	1-2	Wet	Negative	2	34
61	Male	5-10	Wet	Negative	2	32
62	Female	2-5	Wet	Negative	3	26
63	Male	2-5	Wet	Negative	3	35
64	Male	5-10	Wet	Negative	3	35
65	Female	5-10	Wet	Negative	3	33
66	Male	2-5	Wet	Negative	3	35
67	Male	>10	Wet	Negative	1	34
68	Female	>10	Wet	Negative	3	32
69	Male	5-10	Wet	Negative	3	26
70	Male	5-10	Wet	Negative	3	36
71	Male	5-10	Wet	Positive	3	36
72	Female	5-10	Wet	Negative	3	35
73	Male	2-5	Wet	Negative	3	33
74	Female	2-5	Wet	Negative	3	28
75	Female	2-5	Wet	Negative	3	35
76	Female	5-10	Wet	Negative	2	34
77	Female	0-1	Wet	Negative	2	32
78	Male	>10	Wet	Negative	3	28
79	Female	2-5	Wet	Negative	3	35
80	Female	5-10	Wet	Negative	3	34
81	Female	1-2	Wet	Negative	3	32
82	Male	1-2	Wet	Negative	3	26
83	Male	2-5	Wet	Negative	2	36
84	Male	0-1	Wet	Negative	2	36
85	Female	5-10	Wet	Negative	1	35
86	Female	5-10	Wet	Negative	3	33
87	Female	0-1	Wet	Negative	3	35
88	Female	2-5	Wet	Negative	3	34

89	Male	1-2	Wet	Negative	3	32
90	Male	5-10	Wet	Negative	3	28
91	Male	0-1	Wet	Negative	3	35
92	Female	2-5	Wet	Negative	3	34
93	Female	5-10	Wet	Negative	3	35
94	Female	1-2	Wet	Negative	2	34
95	Male	>10	Wet	Negative	2	32
96	Male	2-5	Wet	Negative	3	26
97	Female	5-10	Wet	Negative	3	35
98	Male	0-1	Wet	Negative	3	33
99	Male	>10	Wet	Negative	3	36
100	Male	2-5	Wet	Negative	3	35
101	Male	0-1	Wet	Negative	3	33
102	Male	2-5	Wet	Negative	3	34
103	Female	>10	Wet	Negative	1	32
104	Male	0-1	Wet	Negative	3	28
105	Female	5-10	Wet	Negative	3	35
106	Female	2-5	Wet	Negative	3	34
107	Male	5-10	Wet	Negative	3	35
108	Female	2-5	Wet	Negative	3	34
109	Female	2-5	Wet	Negative	3	32
110	Male	>10	Wet	Negative	3	26
111	Female	2-5	Wet	Negative	3	35
112	Female	5-10	Wet	Negative	3	28
113	Female	2-5	Wet	Negative	3	36
114	Female	1-2	Wet	Negative	2	33
115	Female	1-2	Wet	Positive	3	35
116	Female	1-2	Wet	Negative	3	33
117	Female	0-1	Wet	Negative	3	28
118	Female	5-10	Wet	Negative	3	35
119	Male	0-1	Wet	Negative	3	34
120	Female	>10	Wet	Negative	2	35
121	Male	>10	Wet	Negative	2	28
122	Male	0-1	Wet	Negative	2	33
123	Male	5-10	Wet	Negative	2	34
124	Female	>10	Wet	Negative	3	36
125	Female	0-1	Wet	Negative	2	30
126	Male	5-10	Wet	Negative	2	34
127	Male	2-5	Wet	Negative	2	36
128	Female	>10	Wet	Negative	2	36
129	Male	0-1	Wet	Negative	3	35
130	Female	2-5	Wet	Negative	2	36
131	Male	0-1	Wet	Negative	2	24
132	Female	1-2	Wet	Negative	2	33
133	Female	5-10	Wet	Negative	2	33
134	Female	2-5	Wet	Negative	2	32
135	Male	1-2	Wet	Negative	3	36
136	Male	1-2	Wet	Negative	3	33
137	Female	0-1	Wet	Negative	3	36
138	Male	0-1	Wet	Negative	2	35
139	Male	2-5	Wet	Negative	2	33
140	Male	5-10	Wet	Negative	2	34
141	Female	5-10	Wet	Negative	3	32
142	Male	5-10	Wet	Negative	3	28
143	Male	5-10	Wet	Negative	3	35
144	Female	5-10	Wet	Negative	3	34
145	Female	5-10	Wet	Negative	3	36
146	Female	5-10	Wet	Negative	3	36
147	Male	5-10	Wet	Negative	3	35
148	Male	5-10	Wet	Negative	3	33
149	Male	5-10	Wet	Negative	2	35
150	Male	5-10	Wet	Negative	2	34
151	Male	5-10	Wet	Negative	2	32
152	Female	5-10	Wet	Negative	2	36
153	Male	5-10	Wet	Negative	2	35
154	Male	0-1	Wet	Negative	2	33
155	Female	1-2	Wet	Negative	2	36
156	Male	1-2	Wet	Negative	3	36
157	Male	>10	Wet	Negative	2	35
158	Male	>10	Wet	Negative	1	33
159	Male	5-10	Wet	Negative	1	34
160	Male	5-10	Wet	Negative	3	35
161	Female	5-10	Wet	Negative	3	34
162	Female	5-10	Wet	Negative	3	32
163	Female	1-2	Wet	Negative	3	26
164	Male	5-10	Wet	Negative	3	35
165	Female	1-2	Wet	Negative	3	28
166	Female	1-2	Wet	Negative	3	33
167	Male	5-10	Wet	Negative	3	35
168	Male	5-10	Wet	Negative	3	34
169	Female	0-1	Wet	Negative	3	32
170	Female	5-10	Wet	Negative	2	36
171	Male	1-2	Wet	Negative	2	35
172	Male	2-5	Wet	Negative	2	33
173	Male	5-10	Wet	Negative	2	36
174	Male	5-10	Wet	Negative	2	35
175	Female	0-1	Wet	Negative	2	33
176	Male	5-10	Wet	Negative	1	28
177	Male	5-10	Wet	Negative	3	35
178	Female	0-1	Wet	Negative	3	33
179	Female	0-1	Wet	Negative	3	35
180	Male	0-1	Wet	Positive	3	28
